# Supplementary material for: The evolutionary modifications of a GoLoco motif in the AGS protein facilitate micromere formation in the sea urchin embryo
Source: eLife. 2024 Dec 23;13:RP100086. doi: 10.7554/eLife.100086 (PMC11666239; doi:10.7554/eLife.100086)
Supplement: Figure 7—figure supplement 1—source data 1. [file elife-100086-fig7-figsupp1-data1.zip › Figure 7_Supplement 1B_Source Data 1/Figure7_Supplement1B_Source Data 2.pdf]

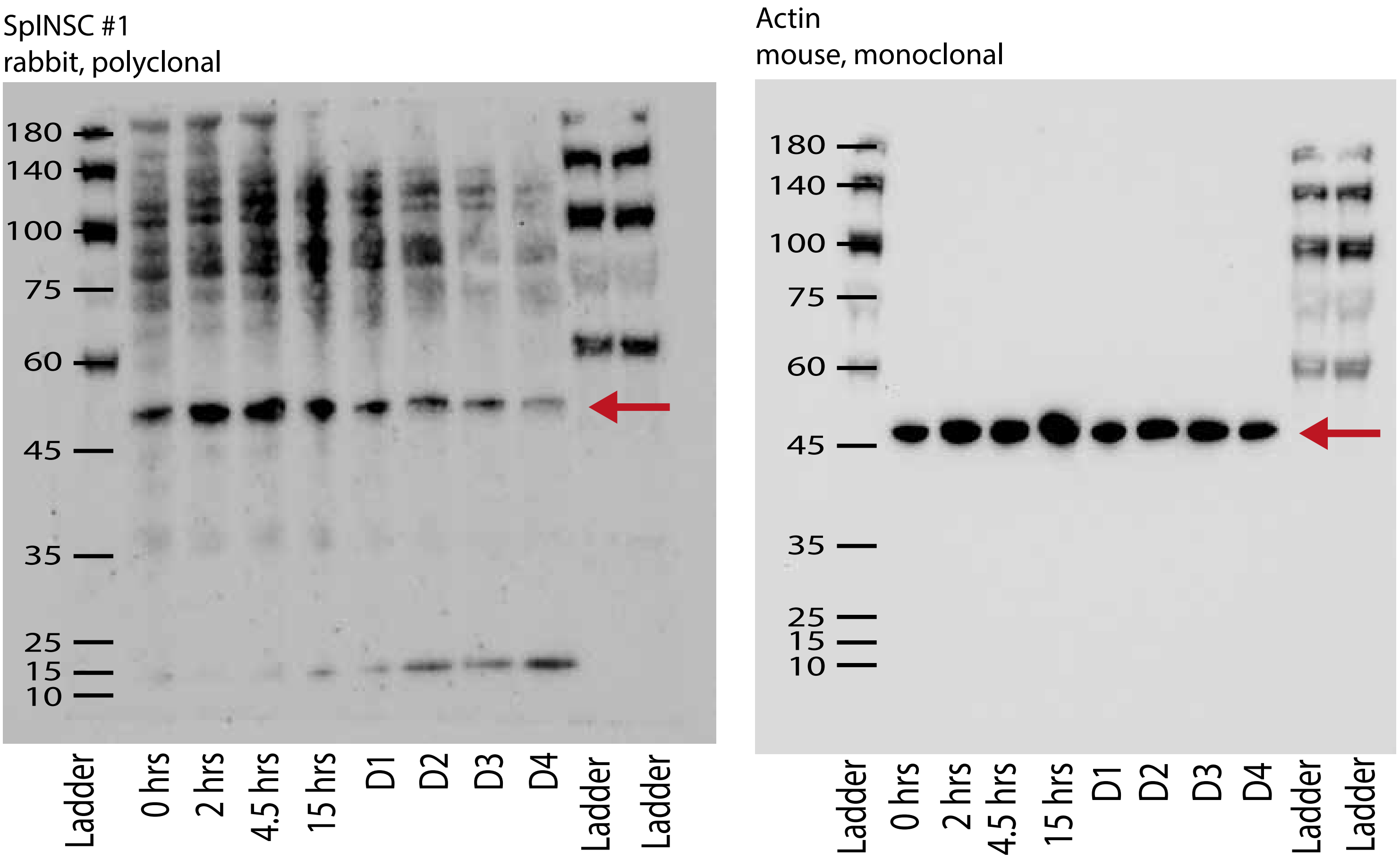

Figure 7 - Supplement 1B, Source Data 2. Original blots corresonding to Figure 7 - Supplement 1B. SpInsc is depicted on the left blot and Actin on the right blot. Red arrows indicate the main expected size.
