# Supplementary figures and images for: The evolutionary modifications of a GoLoco motif in the AGS protein facilitate micromere formation in the sea urchin embryo
Source: eLife. 2024 Dec 23;13:RP100086. doi: 10.7554/eLife.100086 (PMC11666239; doi:10.7554/eLife.100086)

IB from 3-19-21  
Running: 200V, 170mA, 25 min.  
Transfer using iBlot machine

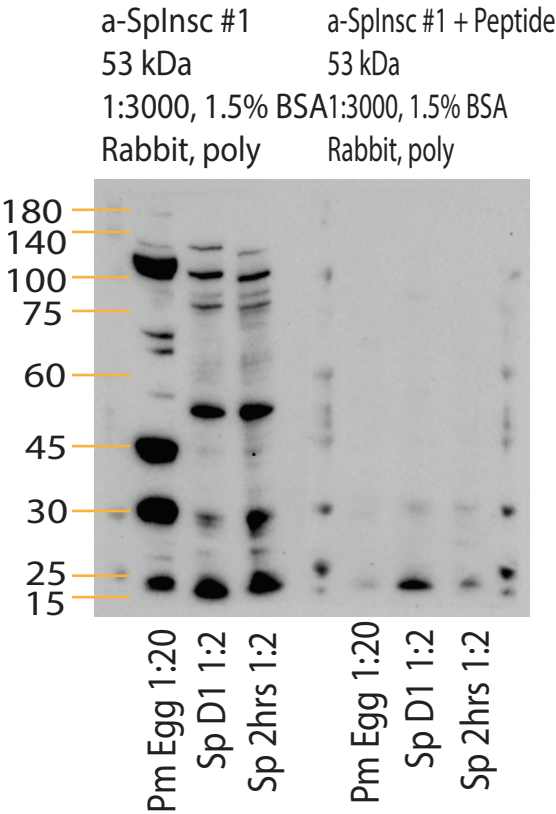

Supplement: Figure 7—figure supplement 1—source data 1. [file elife-100086-fig7-figsupp1-data1.zip › Figure 7_Supplement 1C_Source Data 1/Figure7_Supplement1C_Source Data 2.pdf]

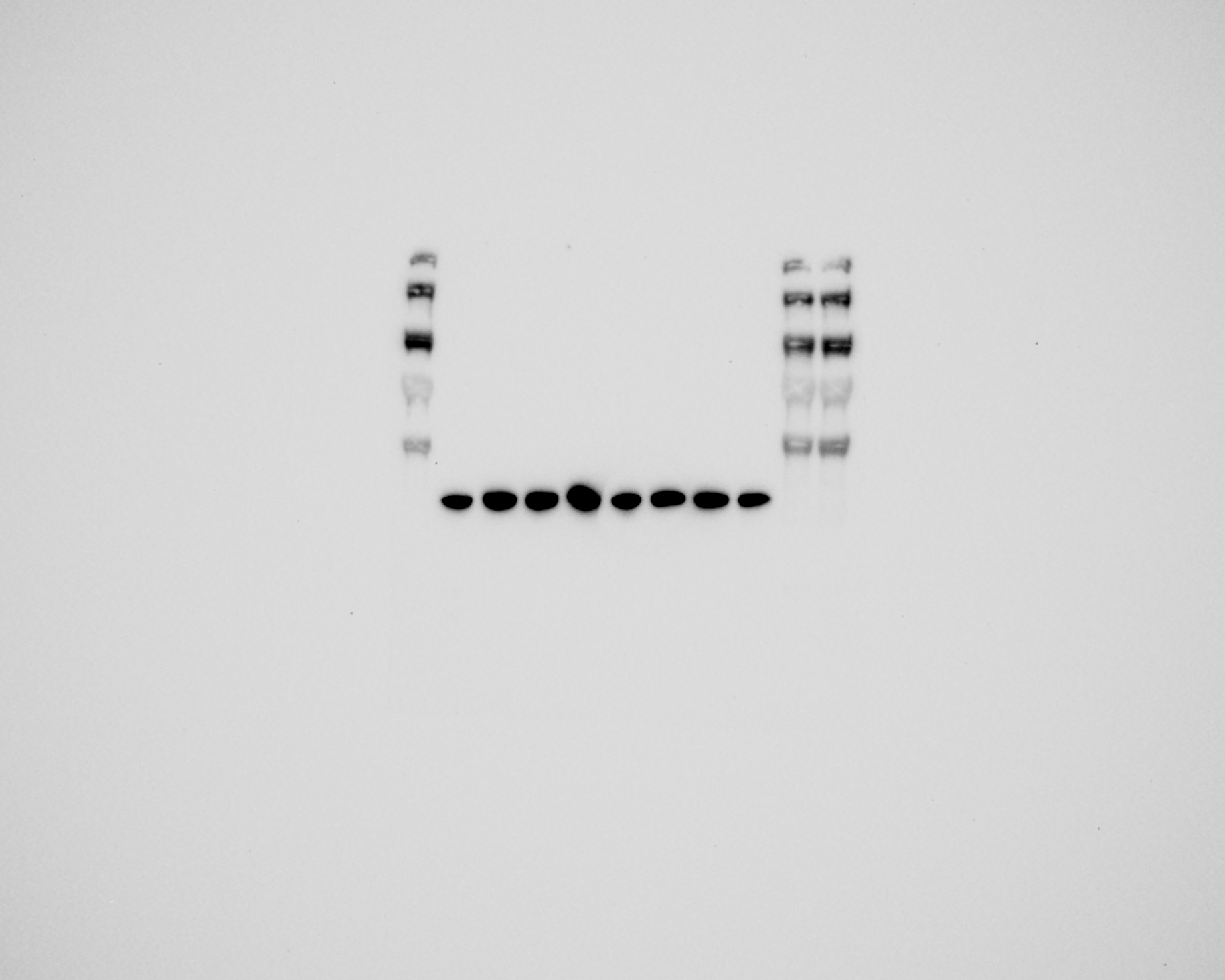

Supplement: Figure 7—figure supplement 1—source data 2. [file elife-100086-fig7-figsupp1-data2.zip › Figure 7_Supplement 1B_Source Data 2/Actin 2020-11-24 12h01m38s(Chemiluminescence).tif]

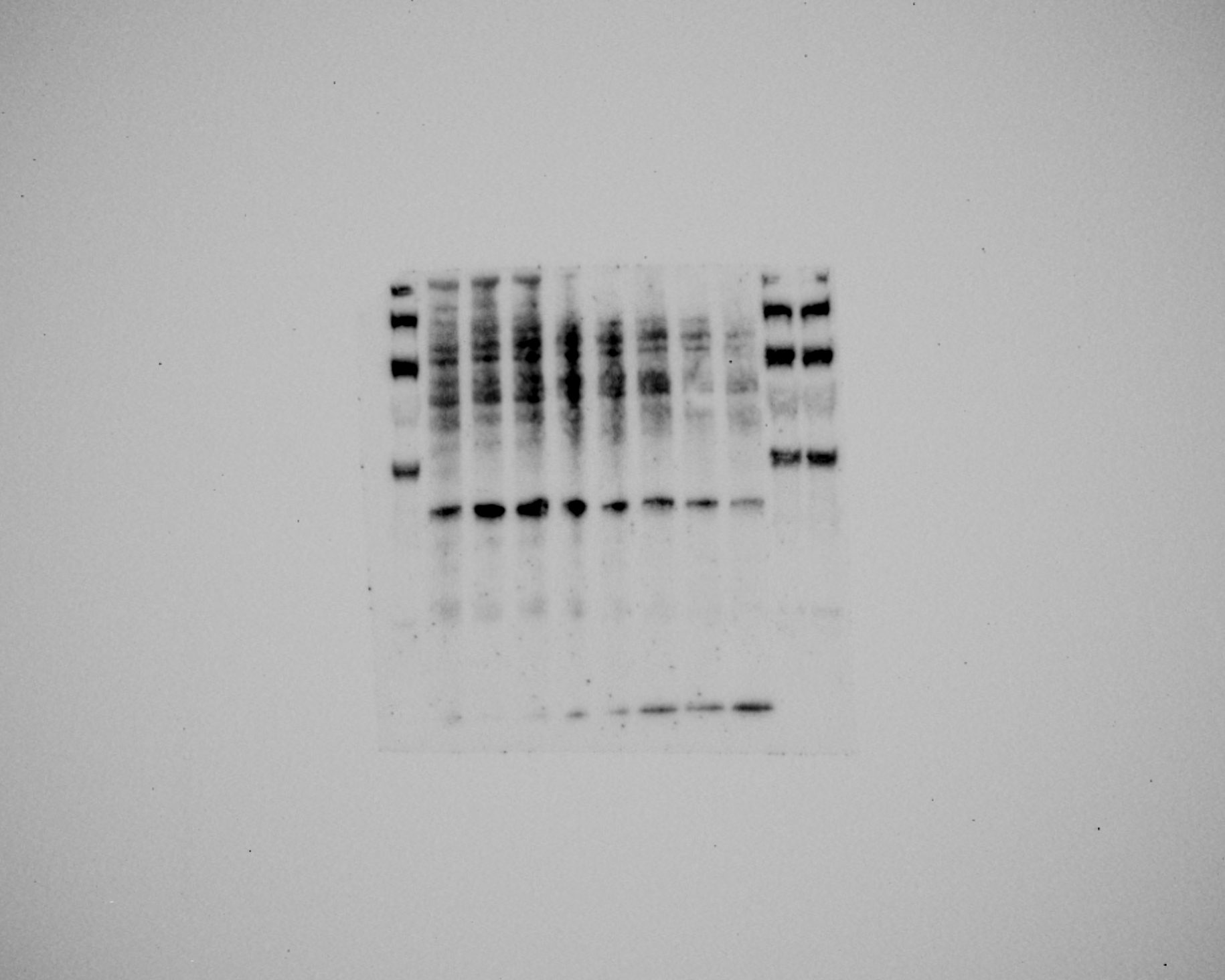

Supplement: Figure 7—figure supplement 1—source data 2. [file elife-100086-fig7-figsupp1-data2.zip › Figure 7_Supplement 1B_Source Data 2/SpINSC_1 2020-11-24 11h57m53s(Chemiluminescence).tif]

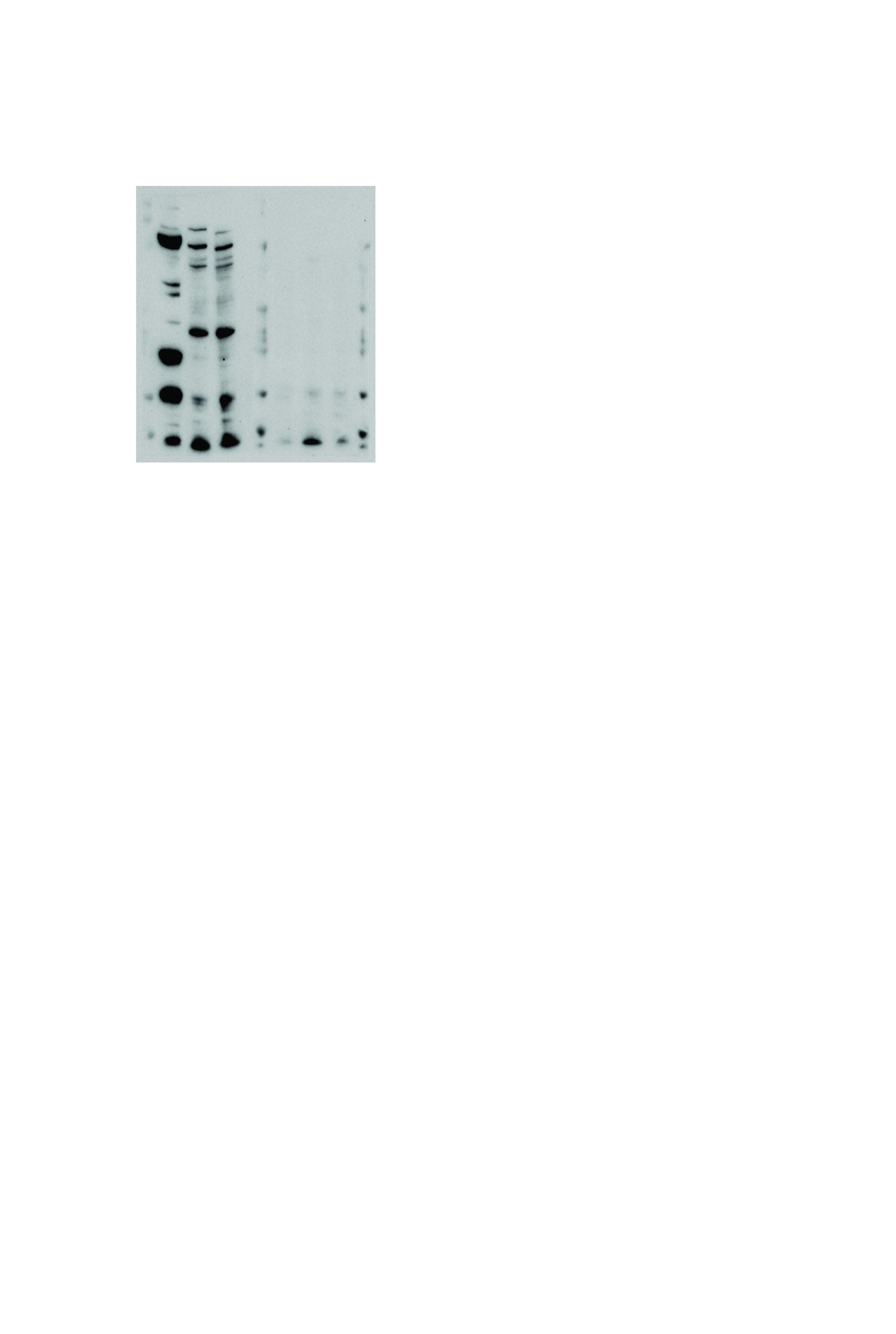

Supplement: Figure 7—figure supplement 1—source data 2. [file elife-100086-fig7-figsupp1-data2.zip › Figure 7_Supplement 1C_Source Data 2/Figure7_Supplement1C_Source Data 1.tiff]
