# Supplementary material for: The evolutionary modifications of a GoLoco motif in the AGS protein facilitate micromere formation in the sea urchin embryo
Source: eLife. 2024 Dec 23;13:RP100086. doi: 10.7554/eLife.100086 (PMC11666239; doi:10.7554/eLife.100086)

IB from 3-19-21
Running: 200V, 170mA, 25 min.
Transfer using iBlot machine

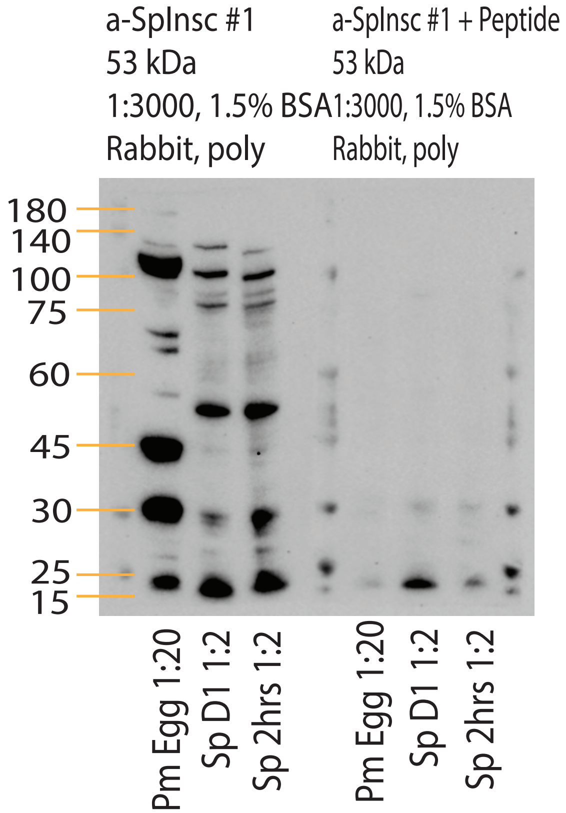

Supplement: Figure 7—figure supplement 1—source data 3. [file elife-100086-fig7-figsupp1-data3.pdf]
